# Appraisal of partial anomalous pulmonary venous drainage through a lumped-parameter mathematical model: a new pathophysiological proof of concept

**DOI:** 10.1093/icvts/ivae175

**Published:** 2024-10-22

**Authors:** Paolo Ferrero, Andrea Tonini, Giulio Valenti, Massimo Chessa, Luca Kuthi, Pier Paolo Bassareo, Luca Dede, Alfio Quarteroni

**Affiliations:** ACHD Unit, Pediatric and Adult Congenital Centre, IRCCS-Policlinico San Donato, San Donato Milanese, Italy; USD Pediatric Cardiology, Azienda Ospedaliera Universitaria Integrata, Verona, Italy; MOX-Modeling and Scientific Computing, Mathematics Department, Politecnico di Milano, Milan, Italy; MOX-Modeling and Scientific Computing, Mathematics Department, Politecnico di Milano, Milan, Italy; ACHD Unit, Pediatric and Adult Congenital Centre, IRCCS-Policlinico San Donato, San Donato Milanese, Italy; Vita-Salute San Raffaele University, Milan, Italy; Heart Center, Semmelweis University, Budapest, Hungary; University College of Dublin, School of Medicine and Mater, Misericordiae University Hospital, Dublin, Ireland; MOX-Modeling and Scientific Computing, Mathematics Department, Politecnico di Milano, Milan, Italy; MOX-Modeling and Scientific Computing, Mathematics Department, Politecnico di Milano, Milan, Italy

**Keywords:** Pulmonary drainage, Anomalous, Model, Ratio between pulmonary and systemic flow

## Abstract

**OBJECTIVES:**

Haemodynamic determinants of the ratio between pulmonary and systemic flow (Qp/Qs) in partial anomalous pulmonary venous return (PAPVR) are still not fully understood. Indeed, among patients with the same number of lung segments draining anomalously, a great variability is observed in terms of right ventricular overload. The aim of this study was to test the hypothesis that the anatomic site of drainage, affecting the total circuit impedance, independently influences the magnitude of shunt estimated by Qp/Qs. A zero-dimensional lumped parameter mathematical model was developed and validated on a sample of patients

**METHODS:**

We developed a zero-dimensional lumped parameter model, using time-varying elastances for heart chambers, RLC Windkessel circuits for the systemic and pulmonary circulations. Patients were categorized into vena cava (VC) type (including left drainage to anomalous vein) and right atrium (RA) type. The mathematical model is a system of ordinary differential equations that are numerically solved by means of the ode15s solver in the MATLAB environment.

**RESULTS:**

The model showed an increase of Qp/Qs with the increase of the number of anomalous veins. With the same number of anomalous veins, Qp/Qs was lower in patients with anomalous drainage to the VC as compared with RA. The validation sample consisted of 49 patients (27, 55% females). As predicted by the model, patients with PAPVR with VC type displayed a lower invasive and cardiac magnetic resonance Qp/Qs as compared with drainage to RA: 1.4 (1.2–1.7) and 1.45 (1.25–1.6) versus 2 (1.75–2.1) and 1.9 (1.6–2), *P* < 0.05. After stratifying for number of lung territories, a lower Qp/Qs was measured in patients with VC PAPVR as compared with RA.

**CONCLUSIONS:**

In patients with PAPVR, the site of anomalous drainage modulates the Qp/Qs. According to the model, this effect is mediated by the post-capillary impedance of the circuit and significantly decreases with the increase of pulmonary vascular resistances.

## Graphical Abstract

**Figure:**
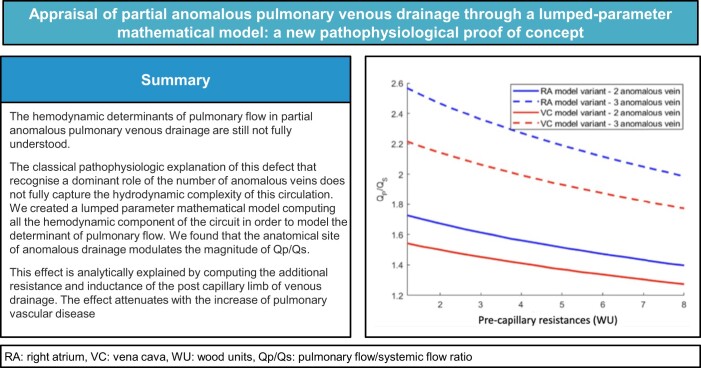

## INTRODUCTION

Partial anomalous pulmonary venous return (PAPVR) represents 0.4–0.7% of congenital heart diseases and is frequently diagnosed in the adult population [1–3]. It reproduces the pathophysiology of pre-tricuspid left-to-right shunt generated by the preferential blood flow to the lower pressure right atrial (RA) chamber through the anomalous veins. However, differently from atrial septal defect, left-to-right shunt is not supposed to be driven by the relative ventricular compliance. Furthermore, contribution of each vein and the effect of the specific anatomic arrangement are not easily predictable. Although there is a high anatomic variability, 2 main categories of variants can be identified: drainage of right-sided pulmonary veins (PVs) into the right atrium (RA) or superior vena cava (SVC) and drainage of left-sided PVs into brachiocephalic vein through a vertical vein. Indication to repair is mainly based on the presence of right ventricular overload on transthoracic echocardiography, cardiac magnetic resonance (CMR) and ratio between pulmonary and systemic flow (Qp/Qs) assessment [2]. Not infrequently, clinicians face patients with comorbidities, equivocal symptoms and with borderline findings, complicating the decision-making algorithm [4, 5]. Therefore, a better understanding of determinants of left-to-right shunt in this defect may help to have a theoretical frame of PAPVR pathophysiology that ultimately might facilitate the interpretation of borderline or conflictual findings. In the classical view, the main determinant of Qp/Qs in PAPVR is the number of lung territories with anomalous drainage. However, among patients with the same number of lung segments draining anomalously, a great variability is observed in terms of right ventricular overload.

The primary objective of this study is to dissect the fluidodynamic mechanisms underlying Qp/Qs variability in PAPVR by using a zero-dimensional (0D), lumped parameters mathematical model. We hypothesized that beside the traditional known factors, the anatomic site of drainage (namely anonymous vein, SVC or RA), by affecting the total circuit impedance, independently influences the magnitude of shunt estimated by Qp/Qs.

The hydrodynamic theoretical plausibility of this hypothesis was verified by using model simulations that were verified in a representative sample of patients.

## METHODS

This paper is structured in a first section in which the PAPVR fluidodynamic is framed by creating an appropriate mathematical model and a second section in which a clinical proof of the concepts revealed by the model is provided. The clinical validation was realized by retrospectively reviewing all adult patients with PAPVR and intact interatrial septum followed in our centre who had undergone either cardiac catheterization or CMR. Qp/Qs was estimated invasively performing a saturation run, taking particular care to obtain a representative sample of mixed venous blood proximal to the shunt. CMR estimation of shunt fraction was calculated by acquisition of phase-contrast velocity flow maps as previously described [6].

### Mathematical modelling

**Figure 1:**
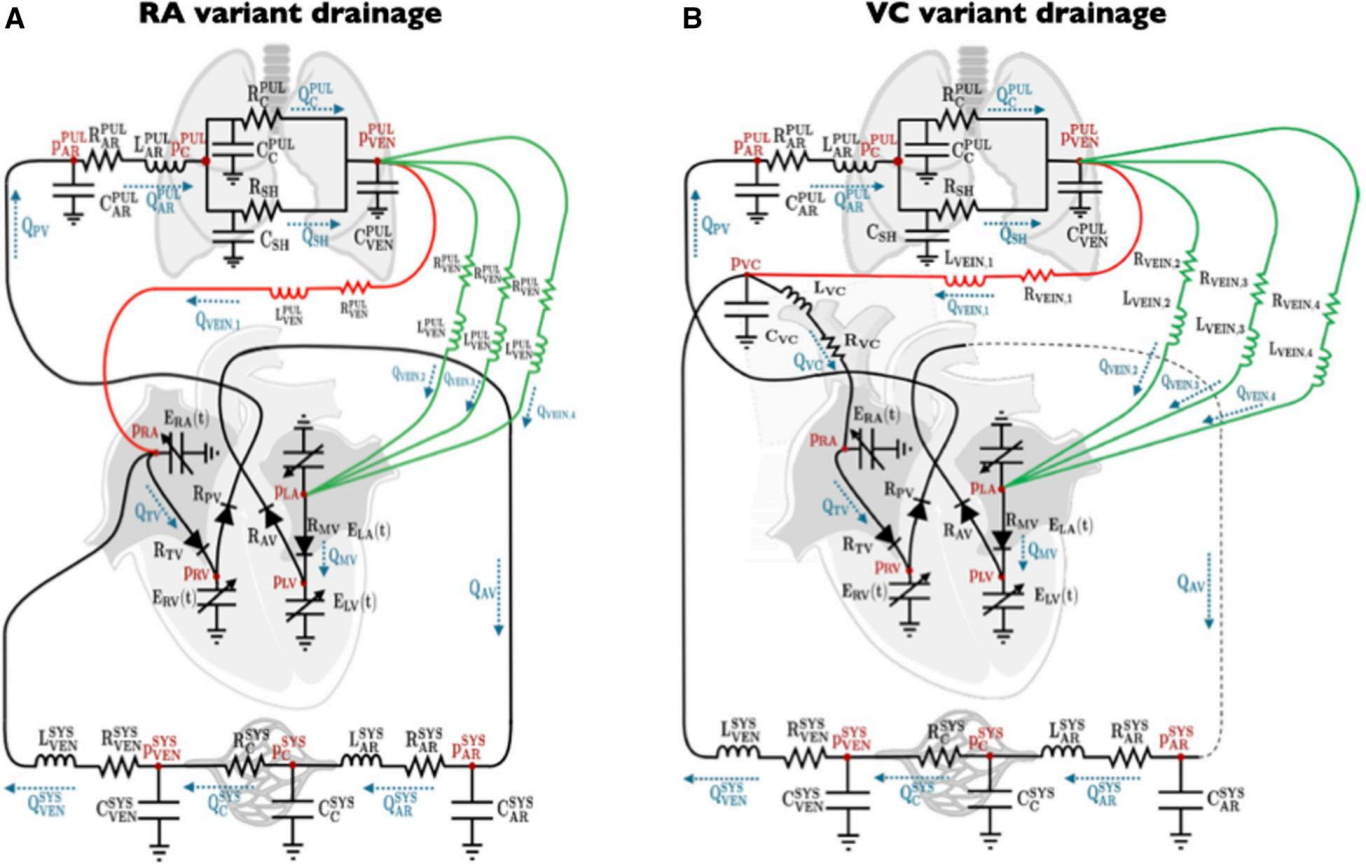
Diagram illustrating clinical varieties of PAPVR (bottom part) with the corresponding circuit analogue of the model (upper part) in the case of drainage in vena cava of a single anomalous vein. PAPVR: partial anomalous pulmonary venous return; RA: right atrium; VC: vena cava.

We developed a 0D cardiovascular model inspired by previous ones present in literature exploiting the electrical analogy [7–9]. We modelled the cardiac chambers as pressure generators by means of time-varying elastances ($E_{LA}\left(t\right),E_{RA}\left(t\right),E_{LV}(t)$ and $E_{RV}(t)$ referring to the left and right atria and to the left and right ventricles, respectively) that mimic the periodic contraction of the cardiac chambers. The valves are modelled as non-ideal diodes ($R_{MV},R_{AV},R_{TV}$ and $R_{PV}$ referring to the mitral, aortic, tricuspid and pulmonary valves, respectively). We split the circulatory system into several compartments, each of them represented by a Windkessel circuit: we subdivided systemic (SYS) and pulmonary (PUL) circulations into arterial (AR), venous (VEN) and capillary (C) compartments that, only in the pulmonary circulation, are further divided into the oxygenated and non-oxygenated ones (SH). The Windkessel circuits describe each compartment by means of one or several components among resistors (R), which represent the resistance to blood flow, capacitances (C), which represent the vessels compliance, and inductances (L), which represent blood inertia. In particular, we modelled each of the 4 PVs with a resistor ($R_{\mathrm{VEIN},1},$ etc.) and an inductance ($L_{\mathrm{VEIN},1},$ etc), together with a common capacitance ($C_{\mathrm{VEN}}^{\mathrm{PUL}}$). For each compartment, the 0D model can provide blood pressures (p) and flows (Q). We assumed normal low pulmonary vascular precapillary resistances, comparable to post-capillary ones, and that the distribution of total lung capillary bed was balanced among the 4 PVs. In order to compute the role of post capillary limb of the circuit, we defined 2 theoretical variants of the model: the RA and vena cava (VC) variant schematized by 1 or more veins connected directly to the RA or upstream to the resistance representing the VC, respectively. In the latter case, we included an additional compartment accounting for both the inferior and SVC (Fig. 1).

### Clinical categorization

Baseline clinical, imaging and catheterization data of adult patients with PAPVR in our centre database were retrospectively reviewed. All patients older than 16 years at the time of 1st encounter, with an anomalous drainage of at least 1 vein and with a valid Qp/Qs estimation were included. Patients with scimitar syndrome, total anomalous venous drainage, associated atrial septal defects and PVR >3 WU were excluded. Possible anatomic arrangements were categorized into 2 main clinical types: right pulmonary venous drainage to superior or inferior VC and RA that we called R variant or left drainage to brachiocephalic vein through a vertical vein, called L variant. To harmonize the clinical categorization with the model, patients were also dichotomized into RA group, which includes the subset of clinical R variant with drainage directly to the RA, and VC group, which includes the subset of clinical R variant with drainage >2 cm distant from RA and clinical L variant. Given the variability of number of veins and extension of lung territory drained by each vein, we have considered 3 territories in the right lung (apical, medium and lower) and 3 in the left (including the lingula), according to the standard functional lung anatomy. Qp/Qs ratio were retrieved for all patients.

### Statistical analysis

Continuous variables are expressed as median and interquartile range and compared using Wilcoxon rank sum and Kruskal–Wallis rank test having observed a clearly skewed distribution by visually inspecting histograms. Categorical variables are presented as counts and percentages. Correlation between quantitative variables was assessed by Pearson r index and Sperman test used to estimate correlation with ranks. A *P* value <0.05 was assumed as statistical significance cut-off.

## RESULTS

### Numerical simulations by zero-dimensional computational model

**Figure 2:**
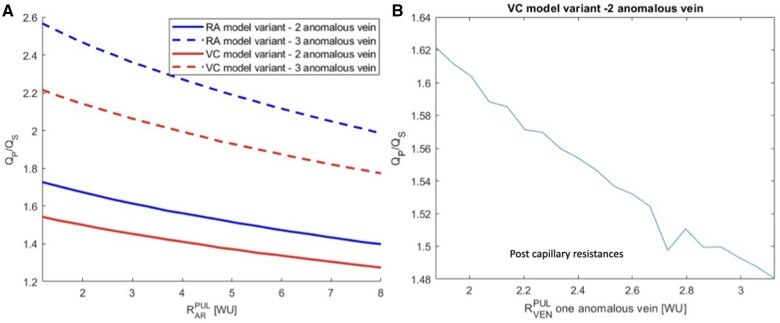
Relation between Qp/Qs and pulmonary resistances predicted by the model according to the site of drainage and number of anomalous veins. Qp/Qs: pulmonary flow/systemic flow ratio; RA: right atrium; VC: vena cava.

The results of the numerical simulations of the 0D model predicted a higher Qp/Qs in RA variant of PAPVR as compared with VC variant. Within each variant, the model predicted a nonlinear increase of Qp/Qs with the increase of the number of anomalous veins. Drainage into the RA produced a higher Qp/Qs as compared to drainage into the VC with the same number of anomalous veins. Indeed, in patients with 1, 2 or 3 veins draining into the RA, the expected Qp/Qs was 1.3, 1.7 and 2.6, respectively, as compared with 1.2, 1.5 and 2.2 for the VC variant (Table 2). The magnitude of this difference was expected to decrease with the increase of the pulmonary resistances. After simulating a gradual increase in the precapillary pulmonary vascular resistances up to 8 WU, the Qp/Qs difference between the RA and VC variant decreased from 0.2 to 0.1 for the case of 2 veins and from 0.4 to 0.2 for the case of 3 veins. More in general, there was an inverse linear relation between Qp/Qs and pulmonary resistances, whatever the number of anomalous veins. On the other hand, by simulating an increase of the resistance of the postcapillary anomalous venous limb, a reduction of the Qp/Qs was predicted by the model (Fig. 2).

**Table 1:** Demographic and haemodynamic data for the whole cohort

*N* = 49	
Female, *n* (%)	27 (55)
Age, median (IQR)	39 (31–52)
Right PAPVR, *n* (%)	36 (73)
Left PAPVR, *n* (%)	13 (27)
Invasive Qp/Qs, median (IQR)	1.65 (1.2–2)
CMR Qp/Qs, median (IQR)	1.5 (1.3–1.9)
PA pressure (mmHg), median (IQR)	16 (8–18)
PVR (mmHg), median (IQR)	1.1 (0.5–1.2)
RA pressure (mmHg), median (IQR)	8 (2–10)

CMR: cardiac magnetic resonance; IQR: interquartile range; PAPVR: pulmonary anomalous pulmonary veins return; PVR: pulmonary vascular resistances; Qp/Qs: ratio between pulmonary and systemic flow; RA: right atrium.

**Table 2:** Catheterization, cardiac magnetic resonance (CMR) and model-based Qp/Qs according to different drainage varieties

Catheterization							
Lung territory	*N* (%)	Clinical variant	*N* (%)	Model variant	*N* (%)	Qp/Qs, median (IQR)	PVR
1	5 (14)	SVC	1 (60)	VC	4 (80)	1.35 (1.2–1.55)	1 (1–1.1)
		RA	1 (20)	RA	1 (20)	1.2	1
		L	3 (20)				
2	17 (47)	SVC	10 (59)	VC	13 (76)	1.4 (1.2–1.4)	1.2 (0.5–1.6)
		RA	4 (23)	RA	4 (24)	2 (1.8–2)	1.4 (1–1.6)
		L	3 (18)				
3	14 (39)	SVC	3 (21)	VC	7 (50)	1.7 (1.3–2)	1.15 (0.7–1.25)
		RA	7 (50)	RA	7 (50)	2 (1.8–2.3)	0.9 (0.7–1.1)
		L	4 (29)				

CMR						
Lung territory	*N* (%)	Clinical variant	*N* (%)	Model variant	*N* (%)	Qp/Qs, median (IQR)
1	7 (29)	SVC	2 (14)	VC	6 (86)	1.3 (1.3–1.6)
		RA	1 (10)	RA	1 (14)	2
		L	4 (57)			
2	16 (47)	SVC	9 (56)	VC	11 (69)	1.5 (1.2–1.6)
		RA	3 (19)	RA	5 (11)	1.5 (1.5–2)
		L	4 (25)			
3	11 (32)	SVC	3 (27)	VC	3 (27)	1.5 (1.5–1.6)
		RA	6 (54)	RA	8(73)	1.9 (1.8–2.2)
		L	2 (18)			

Model			
Lung territory	Model variant	*N* (%)	Qp/Qs, median (IQR)
1	VC		1.2
	RA		1.3
2	VC		1.5
	RA		1.7
3	VC		2.2
	RA		2.6

L: left; PVR: pulmonary vascular resistances; Qp/Qs: ratio between pulmonary and systemic flow; RA: right atrium; SVC: superior vena cava; VC: vena cava.

### Clinical findings

**Figure 3:**
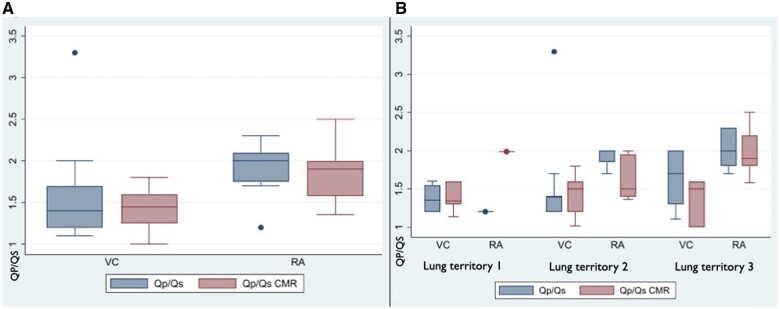
Catheterization and cardiac magnetic resonance estimated Qp/Qs according to different site of venous drainage contemplated by the model. CMR: cardiac magnetic resonance; Qp/Qs: ratio between pulmonary and systemic flow; RA: right atrium; VC: vena cava.

Demographic and haemodynamic data for the whole cohort and according to the different anatomic variants are presented in Tables 1 and 2. Overall, 49 patients (27, 55% females) were included. In the whole cohort, median age was 39 (31–52) years. Thirty-six patients (73%) had the R variant, while 13 (27%) had L variant. Amongst patients with R variant, 4 (11%), 19 (53%) and 13 (36%) had 1, 2 and 3 lung territory draining anomalously, respectively. Among patients with L variant, 5 (38%), 4 (31%) and 4 (31%) patients had 1, 2 and all territories draining anomalously, respectively. By grouping the sample according to classification generated by the model, 19 patients (39%) had an anomalous drainage connected directly to the RA, while 30 (61%) had anomalous veins draining into the VC. In 16 patients (33%), only Qp/Qs estimated at catheterization was recorded, in 13 patients (27%), only CMR Qp/Qs and in 20 (41%), both estimations were available. In the whole cohort, median invasive Qp/Qs was 1.65 (1.2–2) and median CMR Qp/Qs was 1.5 (1.3–1.9). There was a good correlation between Qp/Qs estimated by the 2 methods: Spearman’s rank and Pearson correlation coefficient 0.75 and 0.71, respectively, *P* < 0.001. In the whole cohort, patients with an anomalous drainage of single lung territory displayed a lower Qp/Qs as compared with 2 or 3 territories: 1.2 (1.2–1.5) versus 1.4 (1.2–1.7) versus 1.9 (1.7–2), *P* = 0.02. The same trend was observed with the CMR-based Qp/Qs: 1.4 (1.3–1.6) versus 1.5 (1.3–1.7) versus 1.8 (1.6–2.2), *P* = 0.05. Qp/Qs displayed a direct correlation with the number of lung territories draining through anomalous veins: Spearman’s rho for ranks 0.5 and 0.4, *P* = 0.02 and 0.05 for invasive and CMR Qp/Qs, respectively. Patients with anomalous drainage to the VC displayed a lower median invasive and CMR Qp/Qs as compared with drainage to RA: 1.4 (1.2–1.7) and 1.45 (1.25–1.6) versus 2 (1.7–2) and 1.9 (1.6–2), respectively, *P* < 0.05 for both comparisons. After stratifying for number of lung territories, there was a consistent signal of a higher Qp/Qs in patients with anomalous drainage to VC as compared with RA (Table 1, Fig. 3).

## DISCUSSION

Patients with PAPVR without Atrial septal defect (ASD) represent a peculiar subset of patients with a wide range of clinical signs of pulmonary over-circulation. Classical theoretical pathophysiologic understanding of this defect contemplates that the magnitude of the left-to-right shunt mainly depends on the extension of the pulmonary bed with anomalous drainage. However, it has been empirically observed a great variability in terms of signs of pulmonary overflow among patients with the same number of lung segments with anomalous drainage. In particular, it has been reported that the right venous drainage produces a higher Qp/Qs as compared with left anomalous drainage, being equal to the number of anomalous veins [10].

This observation has been explained taking into account a different extension of the pulmonary vascular bed in the left side as compared to the right, ultimately affecting the amount of pulmonary flow returning to the atria. This view implicitly assumes that blood flow through different pulmonary segments tributaries of a PV is almost exclusively guided by the precapillary impedance and that the role of the postcapillary (venous) section is negligible [11]. This assumption may not hold true in all anatomical variants and cannot explain the clinical variability of pulmonary overflow in patients with the same number of lung territories with anomalous drainage (Fig. 1). Furthermore, clinical framing of PAPVR haemodynamic contemplates only the resistive component of the circuit. In our mathematical model, resistive and inductive components for both the precapillary and postcapillary limbs of the circuits were computed. A main assumption of this model is the equality of arteriolar resistances across pulmonary segments draining through different PVs. This assumption has actually both theoretical and experimental basis [12]. The model predicted that the PAPVR variant with the anomalous veins connecting to VC, which includes right side drainage >2 cm from the RA and L clinical subsets, displays a lower Qp/Qs as compared to the variant with drainage to RA or at VC–RA junction. This difference fitted with invasive and non-invasive Qp/Qs measurements in the clinical sample. The same direction of this trend was reproduced in different subgroups characterized by different numbers of anomalous lung territories. This finding is reported only in a descriptive way, since, due to the small numbers, we did not formally test the difference statistical significance. Within the subset of patients with right venous return, it was also observed a dependence of Qp/Qs from the site of drainage distance from the RA. This finding can also be analytically explained with the introduction of an additional resistance and inductance in the postcapillary limb of the anomalous drainage. The consistency of the effect of the postcapillary resistances is further reinforced by the reduction of Qp/Qs estimated when a selective postcapillary resistance increase of the anomalous veins is simulated. Interestingly, according to the model, the difference in terms of Qp/Qs between the RA and VC variants attenuated when the precapillary resistances raised. This behaviour can be explained taking into account that the effect of the postcapillary limb of the circuit becomes relatively negligible when the precapillary resistive component increases. Therefore, as the precapillary resistances increase, the relative extension of the arterial pulmonary vascular bed is expected to become more preponderant and the site of anomalous drainage negligible in modulating the Qp/Qs. Unfortunately, since we excluded patients with pathological PVRs, we cannot empirically verify this model prediction in our cohort.

### Limitations

This is a preliminary proof of concept paper, mainly based on mechanistic and pathophysiologic considerations and has several limitations. As we used a mathematical model, many assumptions and simplifications were made. First, the numerical simulations obtained from the model implicitly assume instantaneous changes of blood circulation, not accounting for chronic adaptation of the vascular biology. Therefore, although we excluded patients with evidence of pulmonary vascular disease, the effect of additional variables besides those computed in the model cannot be excluded. Second, we modelled the inferior and SVC by means of a single compartment of the mathematical model to restrict our study cases. A representation of the VC in 2 distinct compartments could improve analyses in future works. Furthermore, invasive or CMR-based estimations of Qp/Qs that are clearly affected by measurement errors were assumed to quantify the magnitude of left-to-right shunt. The fluid dynamics hypotheses at the basis of the model are restricted to PAPVR with intact interatrial septum and normal PVRs. Therefore, the corresponding patient sample size is necessarily small, limiting the chances to perform statistically robust comparison, in particular among less represented anatomic varieties, such as those with anomalous drainage of a single lung territory. For all these reasons, the differences that we observed in terms of Qp/Qs, although consistent with the model predictions, must be interpreted in the context of their explorative meaning and need to be confirmed by more powered studies.

## CONCLUSIONS

In conclusion, assuming low/normal precapillary resistances, the site of venous drainage plays a clinically relevant role in modulating the Qp/Qs. This observation is analytically predicted by considering the influence of the total impedance offered by the pre- and post-pulmonary limb of pathway between the pulmonary arteries and the RA. This comprehensive pathophysiological picture may guide a more reproducible and rationale interpretation of haemodynamic and image findings. Finally, these data suggest that the 0 dimensional lumped parameter models are a useful tool to analytically explore the complex pathophysiology of congenital heart diseases characterized by shunt circulations.

## Data Availability

All relevant data are within the manuscript and its Supporting Information files.

## ABBREVIATIONS

0D: Zero-dimensional
CMR: Cardiac magnetic resonance
PAPVR: Partial anomalous pulmonary venous return
PV: Pulmonary vein
Qp/Qs: Ratio between pulmonary and systemic flow
RA: Right atrium
VC: Vena cava

## References

[1] Marelli AJ , Mackie AS, Ionescu-Ittu R, Rahme E, Pilote L. Congenital heart disease in the general population: changing prevalence and age distribution. Circulation 2007;115:163–72. 17210844 10.1161/CIRCULATIONAHA.106.627224

[2] Baumgartner H , De Backer J, Babu-Narayan SV, Budts W, Chessa M, Diller GP et al; ESC Scientific Document Group. 2020 ESC Guidelines for the management of adult congenital heart disease. Eur Heart J 2021;42:563–645. 32860028 10.1093/eurheartj/ehaa554

[3] Healey JE Jr . An anatomic survey of anomalous pulmonary veins: their clinical significance. J Thorac Surg 1952;23:433–44. 14928263

[4] Edwin F. Left-sided partial anomalous pulmonary venous connection—should diagnosis lead to surgery? Interact Cardiovasc Thorac Surg 2010;11:847–8. 21097462 10.1510/icvts.2009.231100A

[5] Giamberti A , Chessa M, Chiarello C, Cipriani A, Carotti A, Galletti L et al Congenital Domain of the Italian Society of Cardiac Surgery, Italian survey on cardiac surgery for adults with congenital heart disease: which surgery, where and by whom? Interact Cardiovasc Thorac Surg 2019;29:260–265. 30907407 10.1093/icvts/ivz045

[6] Chai P , Mohiaddin R. How we perform cardiovascular magnetic resonance flow assessment using phase-contrast velocity mapping. J Cardiovasc Magn Reson 2005;7:705–16. 16136862 10.1081/jcmr-65639

[7] Regazzoni F , Salvador M, Africa PC, Fedele M, Dede L, Quarteroni A. A cardiac electromechanical model coupled with a lumped-parameter model for closed-loop blood circulation. J Comput Phys 2022;457:111083.

[8] Dedè L , Regazzoni F, Vergara C, Zunino P, Guglielmo M, Scrofani R et al Modeling the cardiac response to hemodynamic changes associated with COVID-19: a computational study. Math Biosci Eng 2021;18:3364–83. 34198390 10.3934/mbe.2021168

[9] Tonini A , Vergara C, Regazzoni F, Dede' L, Scrofani R, Cogliati C et al A mathematical model to assess the effects of COVID-19 on the cardiocirculatory system. Sci Rep 2024;14:8304. 38594376 10.1038/s41598-024-58849-3 PMC11004160

[10] Hatipoglu S , Almogheer B, Mahon C, Houshmand G, Uygur B, Giblin GT et al Clinical significance of partial anomalous pulmonary venous connections (isolated and atrial septal defect associated) determined by cardiovascular magnetic resonance. Circ Cardiovasc Imaging 2021;14:e012371. 34384233 10.1161/CIRCIMAGING.120.012371

[11] Seller N , Yoo SJ, Grant B, Grosse-Wortmann L. How many versus how much: comprehensive haemodynamic evaluation of partial anomalous pulmonary venous connection by cardiac MRI. Eur Radiol 2018;28:4598–606. 29721685 10.1007/s00330-018-5428-9

[12] Alpert JS , Dexter L, Vieweg WV, Haynes FW, Dalen JE. Anomalous pulmonary venous return with intact atrial septum: diagnosis and pathophysiology. Circulation 1977;56:870–5. 334393 10.1161/01.cir.56.5.870

